# Anticipated time to seek medical advice for possible lung cancer symptoms and barriers to timely presentation in Palestine: a national cross-sectional study

**DOI:** 10.1186/s12885-024-11935-1

**Published:** 2024-02-07

**Authors:** Mohamedraed Elshami, Nawras Sawafta, Ahmad Mansour, Mohammed Alser, Ibrahim Al-Slaibi, Hanan Abukmail, Hanan Shurrab, Shahd Qassem, Faten Darwish Usrof, Malik Alruzayqat, Wafa Aqel, Roba Nairoukh, Rahaf Kittaneh, Yousef Mahmoud Nimer Habes, Obaida Ghanim, Wesam Almajd Aabed, Ola Omar, Motaz Daraghma, Jumana Aljbour, Razan E. M. Elian, Areen Zuhour, Haneen Habes, Mohammed Al-Dadah, Shurouq I. Albarqi, Bettina Bottcher, Nasser Abu-El-Noor

**Affiliations:** 1 Division of Surgical Oncology, Department of Surgery, University Hospitals Cleveland Medical Center, 11100 Euclid Avenue, Lakeside 7100, Cleveland, OH 44106 USA; 2 Ministry of Health, Gaza, Palestine; 3 Faculty of Medicine, Al-Quds University, Jerusalem, Palestine; 4 Ministry of Health, Ramallah, West Bank Palestine; 5 Faculty of Medicine, Islamic University of Gaza, Gaza, Palestine; 6 The United Nations Relief and Works Agency for Palestine Refugees in the Near East (UNRWA), Gaza, Palestine; 7 Almakassed Hospital, Jerusalem, Palestine; 8 International Medical Corps, Gaza, Palestine; 9 Harvard Medical School, Boston, MA USA; 10 Department of Public Health and Primary Care, University of Cambridge, Cambridge, UK; 11 Faculty of Pharmacy, Al-Azhar University of Gaza, Gaza, Palestine; 12 Department of a Medical Laboratory Sciences, Faculty of Health Sciences, Islamic University of Gaza, Gaza City, Palestine; 13 Faculty of Dentistry, Al-Quds University, Jerusalem, Palestine; 14 Faculty of Nursing, An Najah National University, Nablus, Palestine; 15 Faculty of Dentistry, Al Azhar University of Gaza, Gaza, Palestine; 16 Faculty of Medicine, Al Najah National University, Nablus, Palestine; 17 Faculty of Nursing, Islamic University of Gaza, Gaza, Palestine

**Keywords:** Lung cancer, Awareness, Early presentation, Barriers, Early diagnosis, Palestine

## Abstract

**Background:**

Lung cancer (LC) has poor survival outcomes mainly due to diagnosis at late stages. This study explored the anticipated time to seek medical advice for possible LC symptoms and barriers to early presentation in Palestine.

**Methods:**

This cross-sectional study recruited adult participants from hospitals, primary healthcare centers, and public spaces of 11 governorates using convenience sampling. A modified, translated-into-Arabic version of the validated LC awareness measure was used to assess LC symptom awareness, the time needed to seek medical advice and barriers to early presentation.

**Results:**

A total of 4762 participants were included. The proportion that would immediately seek medical advice for possible LC symptoms varied according to the symptoms’ nature. For respiratory symptoms, this ranged from 15.0% for ‘painful cough’ to 37.0% for ‘coughing up blood’. For non-respiratory symptoms, this ranged from ‘4.2% for ‘unexplained loss of appetite’ to 13.8% for ‘changes in the shape of fingers or nails’. Participants with good LC symptom awareness were more likely to seek medical advice within a week of recognizing most LC symptoms. About 13.0% would delay their visit to see a doctor after recognizing an LC symptom. The most reported barriers were emotional with ‘disliking the visit to healthcare facilities’ (59.8%) as the leading barrier.

**Conclusion:**

LC respiratory symptoms were more likely to prompt early seeking of medical advice. Good LC symptom awareness was associated with a higher likelihood of help-seeking within a week. Educational interventions are needed to promote LC awareness and address the perceived barriers to early presentation in low-resource settings, such as Palestine.

## Introduction

Globally, lung cancer (LC) is the leading cause of cancer-related deaths with estimations of 1,796,144 deaths in 2020 [1]. LC is the third most common cancer in Palestine, following breast and colorectal cancers [2]. LC accounts for 7.8% of all recorded cancer cases in West Bank and 7.4% in Gaza Strip [2]. Furthermore, LC was the leading cause of cancer-related fatalities in 2021, accounting for 16.6% of those recorded [2]. High LC-related mortality could be attributed to delayed presentation and diagnosis at advanced stages [3, 4]. Thus, early diagnosis of LC is critical to improve treatment outcomes and to reduce the impact of disease burden [4]. This is particularly critical in low-resource countries like Palestine because of limited treatment options, fragile healthcare systems, and financial constraints [5, 6].

Previous studies have shown that the seriousness and nature of the symptoms were associated with earlier time to seek medical advice for LC symptoms [7–14]. For example, hemoptysis was perceived as an urgent symptom that led to immediate help-seeking [15–19]. A previous study from Nigeria showed that good LC awareness was associated with shorter time intervals to seek medical advice [20]. A recent national study from Palestine demonstrated that 51.8% of the surveyed participants displayed good awareness of LC symptoms [21].

Presentation at advanced stages is linked to various barriers that could be emotional, practical, or service-related [22]. Having a good awareness of the symptoms related to LC is associated with a higher probability of promptly seeking medical advice [23]. This suggests that through the implementation of campaigns aimed at raising awareness, it is possible to diminish the delay in seeking medical advice, ultimately leading to more positive treatment outcomes [22, 23]. To date, no studies have examined the association between LC symptom awareness and seeking medical advice for possible symptoms of LC in Palestine. Therefore, this national study aimed to (i) describe the time interval to seek medical advice for a possible LC symptom among Palestinians, (ii) examine the association between LC symptom awareness and the anticipated time to seek medical advice, and (iii) explore the barriers to early presentation and their association with LC symptom awareness in Palestine.

## Materials and methods

### Study design and population

This national cross-sectional study was conducted from July 16th, 2019 through to March 29th, 2020. Adult Palestinians, who make up 62% of the approximately five million residents in the Gaza Strip and the West Bank and Jerusalem (WBJ), were the target population [24]. There are 16 governorates in Palestine: five in the Gaza Strip and 11 in the WBJ [24]. Participants were recruited from 11 governorates: four in the Gaza Strip and seven in the WBJ.

### Sample size calculation

In 2019, there were 3,108,852 Palestinians who aged 15 years or older in Palestine [25]. We hypothesized that 40% of study participants at most would immediately seek medical advice for a possible LC symptom. With a confidence level of 95.0%, a type I error rate of 5.0% and an absolute error of 1.0%, the minimum required sample size to detect a 40% proportion of immediate help-seeking for a possible LC symptom was 2399 participants.

### Sampling methods

Most patients in Palestine receive free or low-cost healthcare at governmental hospitals and primary healthcare centers [26]. Using convenience sampling, healthy participants (i.e., without diagnosis of cancer) were recruited from governmental hospitals, primary healthcare centers, and public places including mosques, churches, downtown districts, parks, streets, malls, and public transportation stations in 11 governorates. Recruiting participants from different geographic locations was meant to increase the representativeness of the study cohort [21, 27–37].

### Inclusion and exclusion criteria

Adult (≥ 18 years) Palestinian males and females without history of cancer visiting one of the data collection sites were included in this study. Participants were excluded if they held a nationality other than Palestinian, visited an oncology department at the time of data collection, studied or worked in healthcare, and were unable to complete the questionnaire.

### Validation of study questionnaire

A modified version of the LC awareness measure (LCAM), a validated assessment tool for public awareness of LC, was used for data collection. The original LCAM was translated from English into Arabic and then back translated into English. Each step was done by two different bilingual healthcare professionals who had expertise in clinical research and survey design. To ensure content validity, the questionnaire was reviewed by five independent experts who were specialized in thoracic oncology and public health. This was followed by conducting a pilot study (n = 25) to assess the clarity of questions in the Arabic questionnaire. Data collected in the pilot study were not included in the final analysis. Internal consistency was evaluated using Cronbach's Alpha, which reached an acceptable value of 0.85 for the second and third sections in the questionnaire.

### Measurement tool and data collection

The questionnaire comprised three sections. The first section described participant socio-demographic information including age, gender, marital status, employment status, monthly income, educational level, place of residence, having a chronic disease, knowing someone with cancer, site of data collection. The second section assessed the participants’ awareness of 14 LC symptoms based on a 5-point Likert scale (1 = strongly disagree, 2 = disagree, 3 = not sure, 4 = agree, and 5 = strongly-agree). All 14 LC symptoms were adapted from the original LCAM with yes/no/unknown responses converted into the aforementioned Likert scale to minimize the likelihood of answering questions randomly [21, 27–37]. The second section also assessed the anticipated time to seek medical advice if a possible LC symptom was recognized using an open-ended question. The third section explored the potential barriers for early presentation on the basis of the aforementioned 5-point Likert scale. Out of 21 barriers, 10 were adopted from the original LCAM, whereas the remaining were deemed important to be included based on related previous studies [8, 24, 38–40].

The data collection was done utilizing Kobo Toolbox, a safe and user-friendly data collection software for smartphones. It provides a pre-designed data collection form with check boxes and dropdown options [41]. Participants were invited to complete the questionnaire in a face-to-face interview with the presence of the data collectors, who had a background in healthcare and received special training on how to utilize the electronic tool, approach participants, and facilitate their completion of the questionnaire.

### Statistical analysis

Starting from the age of 45, there is a significant increase in the likelihood of developing LC [42]. Consequently, using this cutoff, the continuous variable of age was divided into two groups: 18–44 years and ≥ 45 years. The minimum wage in Palestine is 1450 NIS (about $450). Therefore, the continuous variable of monthly income was categorized using this cutoff into two categories: < 1450 NIS and ≥ 1450 NIS. [43].

Descriptive statistics were utilized to summarize participant characteristics. The continuous variable of age was non-normally distributed and was described using the median and interquartile range (IQR). Categorical variables were described using frequencies and percentages. Comparisons between baseline characteristics of participants recruited from the Gaza Strip versus the WBJ were performed utilizing Kruskal–Wallis or Pearson's Chi-square test if the characteristic was continuous or categorical, respectively.

LC symptoms were categorized into two categories: (i) respiratory and (ii) non-respiratory symptoms. The anticipated time needed to seek medical advice was categorized into four main categories: immediately (less than 24 h), 1–7 days, > 7 days, and never. Frequencies and percentages were used to describe the anticipated time and Pearson's Chi-Square test with Bonferroni correction was utilized to compare it between participants recruited from the Gaza Strip and those recruited from the WBJ. LC symptom awareness was evaluated as previously described [21, 27–37]. Participants' answers with ‘agree’ or ‘strongly agree’ were considered correct; all other answers were considered incorrect. Each correctly identified LC symptom was given one point. LC symptom awareness level was determined based on the number of symptoms recognized, where the total score (ranged from 0 to 14) was calculated and categorized into three categories: poor (0 to 4), fair (5 to 9), and good awareness (10 to 14). Multivariable logistic regression was used to examine the association between LC symptom awareness and anticipated time to seek medical advice for a possible LC symptom. The multivariable analysis adjusted for age group, gender, education, employment status, monthly income, place of residency, marital status, having a chronic disease, knowing someone with cancer, and site of data collection. This model was determined a priori based on previous studies [21, 27–37].

In consistence with the original LCAM [17], barriers to early presentation were categorized into three main categories: emotional, service, and practical barriers. The displayed barriers (i.e., answering with ‘agree’ or ‘strongly agree’) were described using frequencies and percentages. Comparisons in barriers between participants recruited from the Gaza Strip and those recruited from the WBJ were performed using Pearson’s Chi-Square test. The median number of barriers in each category (overall, emotional, service, and practical) was utilized as a cutoff to dichotomize the number of displayed barriers. This was followed by running a multivariable logistic regression to examine the association between LC symptom awareness and displaying at least the median number of barriers in each category. The same aforementioned multivariable model was used in all those analyses.

A complete case analysis was used to handle missing data, which were hypothesized to be missed completely at random. Stata software, version 17.0 (Stata Corp, College Station, Texas, United States) was used in all statistical analyses.

## Results

### Participant characteristics

Of 5174 participants approached, 4817 agreed and completed the questionnaire (response rate = 93.1%). A total of 4762 questionnaires were included in the final analysis; 2742 from the WBJ and 2020 were from the Gaza Strip, while 55 were excluded; 31 had missing data and 24 did not meet the inclusion criteria. Participants from the WBJ were older, gained higher monthly incomes, but had lower education levels, smoked cigarettes or shisha more frequently, and suffered from more frequent chronic diseases than participants from the Gaza Strip (Table 1). However, participants from both the WBJ and the Gaza Strip had a similar likelihood to display good awareness of LC symptoms (50.6% vs. 52.6%).

**Table 1:** Characteristics of study participants

Characteristic	Total (*n* = 4762)	Gaza Strip (*n* = 2020)	WBJ (*n* = 2742)	*p*-value
**Awareness of LC symptoms,** n (%)				
Poor (0–4 symptoms)	615 (13.0)	279 (13.9)	336 (12.3)	0.21
Fair (5–9 symptoms)	1681 (35.3)	718 (35.5)	963 (35.1)	
Good (10–14 symptoms)	2466 (51.7)	1023 (50.6)	1443 (52.6)	
**Age,** median [IQR]	32.0 [24.0, 44.0]	30.0 [24.0, 40.0]	34.0 [24.0, 47.0]	< 0.001
**Age group,** n (%)				< 0.001
18 to 44	3572 (75.0)	1634 (80.9)	1938 (70.7)	
45 or older	1190 (25.0)	386 (19.1)	804 (29.3)	
**Female gender,** n (%)	2618 (55.0)	1086 (53.8)	1532 (55.9)	0.15
**Educational level,** n (%)				
Secondary or below	2375 (49.9)	955 (47.3)	1420 (51.8)	0.002
Post-secondary	2387 (50.1)	1065 (52.7)	1322 (48.2)	
**Occupation,** n (%)				
Unemployed/housewife	2003 (42.1)	970 (48.0)	1033 (37.7)	< 0.001
Employed	2160 (45.4)	814 (40.3)	1346 (49.1)	
Retired	111 (2.3)	46 (2.3)	65 (2.4)	
Student	488 (10.2)	190 (9.4)	298 (10.8)	
**Monthly income ≥ 1450 NIS,** n (%)	3241 (68.1)	683 (33.8)	2558 (93.3)	< 0.001
**Marital status,** n (%)				
Single	1480 (31.1)	641 (31.7)	839 (30.6)	0.07
Married	3117 (65.5)	1323 (65.5)	1794 (65.4)	
Divorced/Widowed	165 (3.5)	56 (2.8)	109 (4.0)	
**Having a chronic disease,** n (%)	1032 (21.7)	313 (15.5)	719 (26.2)	< 0.001
**Knowing someone with cancer,** n (%)	2571 (54.0)	1045 (51.7)	1526 (55.7)	0.007
**Ever smoked,** n (%)				
Cigarettes	1127 (23.7)	417 (20.6)	710 (25.9)	< 0.001
Shisha (waterpipes)	499 (10.5)	142 (7.0)	357 (13.0)	< 0.001
**Site of data collection,** n (%)				
Public Spaces	1920 (40.3)	784 (38.8)	1136 (41.4)	< 0.001
Hospitals	1628 (34.2)	651 (32.2)	977 (35.7)	
Primary healthcare centers	1214 (25.5)	585 (29.0)	629 (22.9)	

*n* number of participants, *IQR* interquartile range, *WBJ* West Bank and Jerusalem, *LC* lung cancer

### Immediate seeking of medical advice for a possible LC symptom

The proportion of study participants who reported that they would immediately seek medical advice for a possible LC respiratory symptom varied based on the nature of the symptom. For respiratory symptoms, it ranged from 15.0% (*n* = 719) for ‘painful cough’ to 37.0% (*n* = 1763) for ‘coughing up blood’ (Table 2). Participants from the WBJ were more likely than participants from the Gaza Strip to immediately visit a doctor when asked about five out of nine respiratory symptoms.

**Table 2:** Summary of the reported time to seek medical advice for a possible lung cancer symptom

Category of symptoms	LC sign/symptom	Total (*n* = 4762) n (%)	Gaza Strip (*n* = 2020) n (%)	WBJ (*n* = 2742) n (%)
**Respiratory symptoms**	**Coughing up blood**			
	Immediately	1763 (37.0)	594 (29.4)	1169 (42.6)^a^
	1–7 days	2695 (56.6)	1289 (63.8)	1406 (51.3)^a^
	> 7 days	95 (2.0)	41 (2.0)	54 (2.0)
	Never	209 (4.4)	96 (4.8)	113 (4.1)
	**Persistent (3 weeks or longer) chest infection**			
	Immediately	1463 (30.7)	423 (20.9)	1040 (37.9)^a^
	1–7 days	2694 (56.6)	1341 (66.4)	1353 (49.3)^a^
	> 7 days	399 (8.4)	175 (8.7)	224 (8.2)3
	Never	206 (4.3)	81 (4.0)	125 (4.6)
	**A cough that does not go away for two or three weeks**			
	Immediately	1387 (29.1)	403 (20.0)	984 (35.9)^a^
	1–7 days	2550 (53.5)	1266 (62.7)	1284 (46.8)^a^
	> 7 days	365 (7.7)	158 (7.8)	207 (7.5)
	Never	460 (9.7)	193 (9.5)	267 (9.8)
	**Persistent shortness of breath**			
	Immediately	1133 (23.8)	460 (22.8)	673 (24.5)
	1–7 days	3127 (65.7)	1370 (67.8)	1757 (64.1)^a^
	> 7 days	226 (4.7)	84 (4.2)	142 (5.2)
	Never	276 (5.8)	106 (5.2)	170 (6.2)
	**Persistent chest pain**			
	Immediately	1093 (22.9)	380 (18.8)	713 (26.0)^a^
	1–7 days	3165 (66.5)	1432 (70.9)	1733 (63.2)^a^
	> 7 days	284 (6.0)	119 (5.9)	165 (6.0)
	Never	220 (4.6)	89 (4.4)	131 (4.8)
	**Worsening or change in an existing cough**			
	Immediately	1084 (22.8)	446 (22.0)	638 (23.3)
	1–7 days	3144 (66.0)	1354 (67.0)	1790 (65.3)
	> 7 days	261 (5.5)	88 (4.4)	173 (6.3)^a^
	Never	273 (5.7)	132 (6.6)	141 (5.1)
	**An ache or pain when breathing**			
	Immediately	1048 (22.0)	419 (20.7)	629 (22.9)
	1–7 days	3242 (68.1)	1406 (69.6)	1836 (67.0)
	> 7 days	182 (3.8)	66 (3.3)	116 (4.2)
	Never	290 (6.1)	129 (6.4)	161 (5.9)
	**Developing an unexplained loud, high-pitched sound when breathing**			
	Immediately	866 (18.2)	336 (16.6)	530 (19.3)
	1–7 days	2916 (61.2)	1326 (65.6)	1590 (58.0)^a^
	> 7 days	251 (5.3)	84 (4.2)	167 (6.1)^a^
	Never	729 (15.3)	274 (13.6)	455 (16.6)^a^
	**Painful cough**			
	Immediately	719 (15.0)	264 (13.1)	455 (16.6)^a^
	1–7 days	3234 (68.0)	1386 (68.6)	1848 (67.4)
	> 7 days	270 (5.7)	108 (5.3)	162 (5.9)
	Never	539 (11.3)	262 (13.0)	277 (10.1)^a^
**Non-respiratory symptoms**	**Changes in the shape of fingers or nail**			
	Immediately	656 (13.8)	251 (12.4)	405 (14.8)
	1–7 days	1937 (40.7)	860 (42.6)	1077 (39.2)
	> 7 days	528 (11.0)	254 (12.6)	274 (10.0)^a^
	Never	1641 (34.5)	655 (32.4)	986 (36.0)^a^
	**Unexplained weight loss**			
	Immediately	576 (12.0)	168 (8.3)	408 (14.9)^a^
	1–7 days	1641 (34.5)	633 (31.3)	1008 (36.8)^a^
	> 7 days	1126 (23.7)	617 (30.5)	509 (18.5)^a^
	Never	1419 (29.8)	602 (29.9)	817 (29.8)
	**Persistent tiredness or lack of energy**			
	Immediately	383 (8.0)	177 (8.8)	206 (7.5)
	1–7 days	2356 (49.5)	972 (48.1)	1384 (50.5)
	> 7 days	596 (12.5)	307 (15.2)	289 (10.5)^a^
	Never	1427 (30.0)	564 (27.9)	863 (31.5)^a^
	**Persistent shoulder pain**			
	Immediately	363 (7.6)	154 (7.6)	209 (7.6)
	1–7 days	2354 (49.4)	940 (46.5)	1414 (51.6)^a^
	> 7 days	480 (10.1)	214 (10.6)	266 (9.7)
	Never	1565 (32.9)	712 (35.3)	853 (31.1)^a^
	**Unexplained loss of appetite**			
	Immediately	202 (4.2)	102 (5.0)	100 (3.6)
	1–7 days	1579 (33.2)	667 (33.0)	912 (33.3)
	> 7 days	672 (14.1)	312 (15.5)	360 (13.1)
	Never	2309 (48.5)	939 (46.5)	1370 (50.0)

*n* number of participants, *WBJ* West Bank and Jerusalem, *LC* lung cancer

^a^Indicates a *p*-value of less than 0.05

For non-respiratory symptoms, the proportion of immediate help-seeking ranged from 4.2% (*n* = 202) for ‘unexplained loss of appetite’ to 13.8% (*n* = 656) for ‘changes in the shape of fingers or nails’. There were no associated differences in the proportion of immediate seeking of medical advice between participants from the WBJ and those from the Gaza Strip except for ‘unexplained weight loss’, where participants from the WBJ were more likely to immediately visit a doctor.

### Association between LC symptom awareness and immediate seeking of medical advice

Compared to participants with poor LC symptom awareness, those who displayed good LC symptom awareness were more likely to report immediate seeking of medical advice for all respiratory symptoms except for ‘persistent shortness of breath’ and ‘persistent chest pain’, where no associated differences were observed (Table 3). There was no association between displaying good LC symptom awareness and seeking immediate medical advice for all non-respiratory symptoms.

**Table 3:** Multivariable logistic regression analyzing the association between immediate seeking of medical advice and awareness of lung cancer symptoms

A) Respiratory symptoms										
**Awareness of LC symptoms**	**Coughing up blood**		**Persistent (3 weeks or longer) chest infection**		**A cough that does not go away for two or three weeks**		**Persistent shortness of breath**		**Persistent chest pain**	
	**AOR (95% CI)**	***p*-value**	**AOR (95% CI)**	***p*-value**	**AOR (95% CI)**	***p*-value**	**AOR (95% CI)**	***p*-value**	**AOR (95% CI)**	***p*-value**
**Poor**	Ref	Ref	Ref	Ref	Ref	Ref	Ref	Ref	Ref	Ref
**Fair**	1.17 (0.95–1.43)	0.14	1.24 (0.99–1.55)	0.06	1.15 (0.92–1.44)	0.23	0.82 (0.66–1.03)	0.09	0.82 (0.66–1.03)	0.10
**Good**	1.80 (1.47–2.19)	< 0.001	1.72 (1.39–2.14)	< 0.001	1.69 (1.36–2.10)	< 0.001	1.13 (0.92–1.39)	0.26	1.19 (0.96–1.48)	0.11
**Awareness of LC symptoms**	**Worsening or change in an existing cough**		**An ache or pain when breathing**		**Developing an unexplained loud, high-pitched sound when breathing**		**Painful cough**			
	**AOR (95% CI)**	***p*-value**	**AOR (95% CI)**	***p*-value**	**AOR (95% CI)**	***p*-value**	**AOR (95% CI)**	***p*-value**		
**Poor**	Ref	Ref	Ref	Ref	Ref	Ref	Ref	Ref		
**Fair**	0.96 (0.76–1.21)	0.73	0.89 (0.70–1.12)	0.31	1.09 (0.83–1.42)	0.53	0.96 (0.72–1.26)	0.75		
**Good**	1.45 (1.16–1.83)	0.001	1.45 (1.16–1.81)	0.001	1.89 (1.47–2.43)	< 0.001	1.53 (1.17–1.99)	0.002		
**B) Non-respiratory symptoms**										
**Awareness of LC symptoms**	**Changes in the shape of fingers or nails**		**Unexplained weight loss**		**Persistent tiredness or lack of energy**		**Persistent shoulder pain**		**Unexplained loss of appetite**	
	**AOR (95% CI)**	***p*-value**	**AOR (95% CI)**	***p*-value**	**AOR (95% CI)**	***p*-value**	**AOR (95% CI)**	***p*-value**	**AOR (95% CI)**	***p*-value**
**Poor**	Ref	Ref	Ref	Ref	Ref	Ref	Ref	Ref	Ref	Ref
**Fair**	0.91 (0.69–1.19)	0.49	0.70 (0.53–0.92)	0.011	0.69 (0.49–0.97)	0.032	0.60 (0.43–0.85)	0.003	0.61 (0.39–0.96)	0.033
**Good**	1.10 (0.85–1.43)	0.45	0.93 (0.72–1.21)	0.60	1.17 (0.86–1.60)	0.32	0.97 (0.72–1.33)	0.87	1.11 (0.74–1.66)	0.61

*AOR* adjusted odds ratio, *CI* confidence interval, *LC* lung cancer

- All analyses were adjusted for age-group, gender, educational level, occupation, monthly income, marital status, residency, having a chronic disease, knowing someone with cancer, smoking history, and site of data collection

- The outcome in all models is binary, where answers with ‘immediately’ were considered as ‘yes’ and all other answers were considered as ‘no’

### Association between LC symptom awareness and seeking medical advice within a week

Compared to participants with poor LC symptom awareness, those who displayed fair LC symptom awareness were more likely to seek medical advice within a week for all LC symptoms except for ‘persistent tiredness or lack of energy’, ‘persistent shoulder pain’, and ‘unexplained loss of appetite’, where no associated differences were observed (Table 4). Additionally, there was a further associated increase in the likelihood of seeking medical advice within a week for all LC symptoms among participants who displayed good LC symptom awareness except for ‘unexplained weight loss,’ where no associated difference was found.

**Table 4:** Multivariable logistic regression analyzing the association between seeking medical advice within a week and awareness of lung cancer symptoms

A) Respiratory symptoms										
**Awareness of LC symptoms**	**Coughing up blood**		**Persistent (3 weeks or longer) chest infection**		**A cough that does not go away for two or three weeks**		**Persistent shortness of breath**		**Persistent chest pain**	
	**AOR (95% CI)**	***p*-value**	**AOR (95% CI)**	***p*-value**	**AOR (95% CI)**	***p*-value**	**AOR (95% CI)**	***p*-value**	**AOR (95% CI)**	***p*-value**
**Poor**	Ref	Ref	Ref	Ref	Ref	Ref	Ref	Ref	Ref	Ref
**Fair**	1.44 (1.05–1.99)	0.026	1.37 (1.06–1.77)	0.016	1.35 (1.07–1.70)	0.010	1.53 (1.17–2.01)	0.002	1.41 (1.07–1.84)	0.013
**Good**	2.45 (1.76–3.42)	< 0.001	1.85 (1.44–2.38)	< 0.001	2.04 (1.63–2.57)	< 0.001	2.32 (1.77–3.03)	< 0.001	2.05 (1.57–2.69)	< 0.001
**Awareness of LC symptoms**	**Worsening or change in an existing cough**		**An ache or pain when breathing**		**Developing an unexplained loud, high-pitched sound when breathing**		**Painful cough**			
	**AOR (95% CI)**	***p*-value**	**AOR (95% CI)**	***p*-value**	**AOR (95% CI)**	***p*-value**	**AOR (95% CI)**	***p*-value**		
**Poor**	Ref	Ref	Ref	Ref	Ref	Ref	Ref	Ref		
**Fair**	1.44 (1.10–1.89)	0.007	1.76 (1.35–2.30)	< 0.001	1.42 (1.15–1.76)	0.001	1.61 (1.28–2.01)	< 0.001		
**Good**	2.10 (1.61–2.75)	< 0.001	2.80 (2.14–3.68)	< 0.001	2.36 (1.90–2.92)	< 0.001	2.17 (1.74–2.72)	< 0.001		
**B) Non-respiratory symptoms**										
**Awareness of LC symptoms**	**Changes in the shape of fingers or nails**		**Unexplained weight loss**		**Persistent tiredness or lack of energy**		**Persistent shoulder pain**		**Unexplained loss of appetite**	
	**AOR (95% CI)**	***p*-value**	**AOR (95% CI)**	***p*-value**	**AOR (95% CI)**	***p*-value**	**AOR (95% CI)**	***p*-value**	**AOR (95% CI)**	***p*-value**
**Poor**	Ref	Ref	Ref	Ref	Ref	Ref	Ref	Ref	Ref	Ref
**Fair**	1.22 (1.01–1.48)	0.038	0.80 (0.66–0.97)	0.023	1.17 (0.97–1.41)	0.11	1.19 (0.99–1.44)	0.07	0.94 (0.77–1.14)	0.53
**Good**	1.56 (1.30–1.87)	< 0.001	1.02 (0.85–1.23)	0.80	1.55 (1.29–1.86)	< 0.001	1.56 (1.30–1.87)	< 0.001	1.24 (1.03–1.50)	0.025

*AOR* adjusted odds ratio, *CI* confidence interval, *LC* lung cancer

- All analyses were adjusted for age-group, gender, educational level, occupation, monthly income, marital status, residency, having a chronic disease, knowing someone with cancer, smoking history, and site of data collection

- The outcome in all models is binary, where answers with ‘immediately’ and ‘1–7 days’ were considered as ‘yes’ and all other answers were considered as ‘no’

### Barriers to early presentation and their association with LC symptom awareness

When participants were asked if they would delay their visit to the doctor when an LC symptom was recognized, 615 (12.9%) answered with ‘yes’ and reported at least one barrier to early presentation. The most frequent emotional barrier was ‘disliking the visit to healthcare facilities’ (*n* = 368, 59.8%) (Table 5). The most frequent service barrier was ‘the thought that there is a small number of qualified doctors in the Palestinian Ministry of Health facilities’ (*n* = 263, 42.8%). The most frequent practical barriers were ‘would think the symptom is not something important to see the doctor’ (*n* = 353, 57.4%) and ‘would try some herbs first’ (*n* = 348, 56.6%). All these findings were consistent in both the Gaza Strip and the WBJ with the exception that the most reported practical barrier in the Gaza Strip was ‘would try some herbs first’ (*n* = 137, 58.3%) followed by ‘financial hardship that will not allow seeing the doctor’ (*n* = 121, 51.5%). On the multivariable analysis, displaying a fair or good awareness of LC symptoms was not associated with reporting all, emotional, service, or practical barriers (Table 6).

**Table 5:** Summary of the reported barriers to early presentation among study participants

Category	Barrier	Total (*n* = 615) n (%)	Gaza Strip (*n* = 235) n (%)	WBJ (*n* = 380) n (%)	*p*-value
**Emotional**	Disliking the visit to healthcare facilities	368 (59.8)	136 (57.9)	232 (61.1)	0.45
	Feeling worried about what a doctor might find	313 (50.9)	118 (50.2)	195 (51.3)	0.80
	God is the ultimate healer so there in no need to go to the doctor	291 (47.3)	95 (40.4)	196 (51.6)	0.008
	Would prefer not to know the reason of the symptom	273 (44.4)	90 (38.3)	183 (48.2)	0.019
	Feeling scared	268 (43.6)	108 (46.0)	160 (42.1)	0.36
	Would not trust the doctor	224 (36.4)	103 (43.8)	121 (31.8)	0.003
	Would not feel comfortable to be examined by a doctor of a different gender from the participant’s	164 (26.7)	75 (31.9)	89 (23.4)	0.024
	Embarrassment	110 (17.9)	46 (19.6)	64 (16.8)	0.39
	Would not feel confident talking about symptom with the doctor	79 (12.9)	33 (14.0)	46 (12.1)	0.54
**Service**	The thought that there is a small number of qualified doctors in the Palestinian Ministry of Health facilities	263 (42.8)	110 (46.8)	153 (40.3)	0.11
	Difficulty making an appointment with the doctor	191 (31.1)	70 (29.8)	121 (31.8)	0.65
	Feeling worried about wasting doctor’s time	150 (24.4)	58 (24.7)	92 (24.2)	0.92
	Difficulty talking to doctor	128 (20.8)	55 (23.4)	73 (19.2)	0.22
**Practical**	Would think that symptom is not something important to see the doctor for	353 (57.4)	117 (49.8)	236 (62.1)	0.003
	Would try some herbs first	348 (56.6)	137 (58.3)	211 (55.5)	0.50
	Has other things to do	282 (45.9)	95 (40.4)	187 (49.2)	0.037
	Too busy to go to the doctor	280 (45.5)	103 (43.8)	177 (46.6)	0.56
	Financial hardship that will not allow seeing the doctor	227 (36.9)	121 (51.5)	106 (27.9)	< 0.001
	Difficulty arranging transports to the doctor	150 (24.4)	86 (36.6)	64 (16.8)	< 0.001
	Family or partner would refuse the participant being examined by a doctor of a different gender	52 (8.5)	24 (10.2)	28 (7.4)	0.23
	Family or partner would refuse the visit to the doctor	44 (7.2)	23 (9.8)	21 (5.5)	0.054

*n* number of participants, *WBJ* West Bank and Jerusalem

**Table 6:** Multivariable logistic regression analyzing the association between lung cancer symptom awareness and having at least the median number of barriers to seek medical advice for a possible lung cancer symptom

Awareness of LC symptoms	All barriers		Emotional barriers		Service barriers		Practical barriers	
	**AOR (95% CI)**^a^	***p*-value**	**AOR (95% CI)**^a^	***p*-value**	**AOR (95% CI)**^a^	***p*-value**	**AOR (95% CI)**^a^	***p*-value**
Poor	Ref	Ref	Ref	Ref	Ref	Ref	Ref	Ref
Fair	1.21 (0.71–2.08)	0.49	1.04 (0.59–1.83)	0.88	0.86 (0.48–1.53)	0.60	1.43 (0.84–2.43)	0.19
Good	1.17 (0.70–1.98)	0.55	1.02 (0.59–1.75)	0.95	0.91 (0.52–1.60)	0.75	1.22 (0.73–2.04)	0.45

*AOR* adjusted odds ratio, *CI* confidence interval, *WBJ* West Bank and Jerusalem, *LC* lung cancer

The outcome in all models is binary with the median number of barriers in each category utilized as the cutoff

^a^Adjusted for age-group, gender, educational level, occupation, monthly income, marital status, residency, having a chronic disease, knowing someone with cancer, smoking history, and site of data collection

## Discussion

In this study, participants would more often seek medical advice earlier if LC respiratory symptoms were recognized compared with non-respiratory symptoms, however, less than half reported immediate help-seeking. Displaying good LC awareness was associated with a higher likelihood of seeking medical advice within a week from recognizing most LC symptoms. About 13% would delay help-seeking after recognizing an LC symptom and displayed different barriers. Individuals' approaches to seeking healthcare services, particularly when faced with LC symptoms, play a pivotal role in determining the timeliness and effectiveness of interventions [22]. Understanding health-seeking behaviors helps identify barriers and facilitators that influence when and how individuals access cancer care [24, 44].

Similar to previous studies from Australia, England, Ireland, and Denmark, ‘coughing up blood’ was one of the most frequently reported respiratory symptoms that led most participants to seek medical advice immediately [18, 19, 24, 45, 46]. Furthermore, previous studies from Palestine showed that patients were more likely to associate their symptoms with cancer if blood is present [27, 37]. This could be explained by the tendency of people to associate bleeding with ominous diagnoses and evidence of cancer [16, 45]. In contrast, ‘painful cough’ prompted immediate help-seeking only by a small proportion of study participants, which comes in concordance with findings of a previous study by Caswell and colleagues [7]. Cough is a common complaint with a wide range of differential diagnosis such as chest infections, seasonal changes, smoking, occupational exposure to environmental hazards, chronic obstructive pulmonary disease, asthma, or tuberculosis. Most participants may have experienced cough previously due to various causes, leading them to suspect possible benign causes to be more likely reasons for their cough [8, 20, 38–40]. A previous study conducted in Palestine reported that ‘worsening in an existing cough’ was the most recognized respiratory LC symptom [21]. Similarly, Shankar and colleagues found that two thirds of participants would seek medical help for persistent cough [47]. This suggests that the public may perceive persistence and change in character of the cough as more ominous signs, which might drive them to seek medical advice earlier. Moffat and colleagues demonstrated the impact of national cancer awareness campaigns for LC on key symptom awareness and showed that the proportion of people recognizing ‘chronic cough’ as a definite warning sign of LC rose from 18% pre-intervention to 33% post-intervention [4]. Therefore, educational interventions should incorporate the nature and character of LC symptoms.

Having good LC symptom awareness is associated with a higher likelihood of seeking medical advice earlier, as evidenced by the UK's national "Be Clear on Cancer" campaign, which significantly increased LC awareness and thus medical advice seeking and achieved better therapeutic outcomes [23]. Our results support this as we found that good LC symptom awareness was generally associated with an increase in the likelihood of seeking medical advice within a week compared to poor awareness. Likewise, a study from Nigeria showed that 76% of participants, who were able to recognize one or more of LC warning signs, would seek medical advice in less than 2 weeks compared to only 24% of those who did not recognize any [20]. Thus, by implementing campaigns that aim to increase public awareness, it appears to be possible to reduce time to seek medical advice and improve LC outcomes [22, 23]. It is also important to mention that there were regional variations in the willingness to seek immediate medical advice among participants from the WBJ versus those from the Gaza Strip, where the former group were more likely to report immediate help-seeking for more than half of LC respiratory symptoms. A possible explanation for this could be the poorer economic circumstances in the Gaza Strip. Approximately 53% of individuals in the Gaza Strip were identified as poor in 2017, reflecting a poverty rate more than four times higher than that of the West Bank and Jerusalem [48].

One of the contributing factors to delays in seeking medical advice is the experience people may have made with the healthcare system [24, 44, 49]. According to a survey conducted in the United States, more than one-third of participants had 'unfavorable evaluations of seeking medical care' due to factors such as problems with doctors and/or healthcare institutions [50]. Moreover, 12% felt that they did not need to seek medical attention as often, because they thought their illness or symptoms will resolve spontaneously [50]. This was also found in this study, where a large proportion reported barriers such as ‘dislike to visit healthcare facilities’ or ‘distrust in the competence of the local healthcare providers’ and ‘would think the symptom is not something important to see the doctor’ as well as ‘would try some herbs first’. Moreover, an Australian study found that participants with an Arabic culture had a lower level of trust in doctors compared to other study groups [16]. This demonstrates that good relationships between healthcare providers and patients may facilitate help-seeking and possibly also overcome mistrust if present [44, 51].

This is especially important among patients who smoke. Although smoking is frequently recognized as an LC risk factor, smokers were found to be less likely to seek medical advice while having possible LC symptoms as they tended to relate them to smoking [52]. Furthermore, beliefs that doctors have negative attitudes towards smokers have been found to impede help-seeking behavior [16, 24, 44, 53–56]. Alleviating these factors should be integrated in future interventions aiming to facilitate effective communication between healthcare providers and patients [55].

### Future directions

The results of this study demonstrate the critical need to improve the public knowledge about LC symptoms and importance of early presentation in Palestine. Educational interventions to improve LC awareness and to address barriers to early presentation are crucial to enhance early presentation and thus improve LC outcomes. Healthcare professionals should encourage early seeking of medical advice in case LC symptoms are recognized. There is also a need for a multidisciplinary and culturally tailored approach to help individuals rationalize their presumptions, distrust and dislike towards healthcare providers. This could probably promote help-seeking and strengthen physician–patient interpersonal relationship.

### Limitations

This study has some limitations. First, the convenience sampling utilized does not guarantee the generalizability of the results. However, the large number of participants, high response rate, and recruitment from various geographic locations may have mitigated this limitation. Second, potential participants with backgrounds in healthcare and patients in the oncology departments were excluded from the study, which might have impacted our interpretations. However, their exclusion was intended to make this study more relevant to the public. Lastly, participants were asked about their anticipated help-seeking behavior while they did not have any of the examined LC symptoms. It is plausible that their response and help-seeking behavior could be altered by actual experience of LC symptoms.

## Conclusion

In this study, LC respiratory symptoms more often prompted immediate help-seeking with coughing up blood as the most alarming symptom. Participants with good LC symptom awareness had a higher likelihood of seeking medical advice within a week of recognizing most LC symptoms. About 13% would delay their doctor visit after recognizing an LC symptom due to various perceived barriers. Educational campaigns are needed to increase public awareness, reduce barriers to early presentation, and make positive impact on health behaviors.

## Data Availability

The dataset used and analyzed during the current study is available from the corresponding author on reasonable request.

## Abbreviations

LC: Lung cancer
MOH: Ministry of Health
WBJ: West Bank and Jerusalem
LCAM: Lung Cancer Awareness Measure
CI: Confidence interval
OR: Odds ratio
